# The Pellicle of an Egg, an Adhesive Application to Wounds

**Published:** 1840

**Authors:** 


*The Pellicle of an Egg, an excellent Adhesive Application to Wounds.*

The following letter from M. Colquet, we find in a late number of the Bulletin Medical Belge:

*To the Editor—*

When reading a notice by Dr. Heusner, in Casper's Wochenscraft, of the employment of the pellicle of an egg as an application to recent wounds, I was reminded of an interesting anecdote, told me by a veteran soldier of Napoleon.

It was at the seige of Saragossa, when the town had been given up to pillage, and the only inhabitants that remained, were concealed in cellars and haylofts. No quarters were shown to any one; men, women and children were butchered without mercy.

The soldier, my informant, was not more compassionate than his comrades. He was, however, moved with pity at the sight of a poor infant, which he found lying under its cradle, and which must have fallen a sacrifice had he not interposed. He took it up in his arms, and was conveying it away to some place of safety, when he received a sabre cut across the face, which nearly severed his nose. The flap hung down upon his upper lip, and the wound bled very profusely.

Fortunately a chemist's shop was near; he went in, and the *pharmacien* treated him with the greatest kindness—"Oh! he was no Spaniard," repeated the old invalid, over and over again, for *he* would have poisoned us. The wound was cleaned, and the detached flap of his nose was brought together: when the bleeding had ceased, he took an egg and broke it, and then separating the pellicle of the shell, he spread this over the nose (en coiffa le nez du soldat.)

At this time the drums beat to quarters; off, therefore, he had to be on the instant; and from that time to the end of the Spanish campaign I forgot, said the old man, all about my nose; and at length when I looked at myself in a glass, "j'etais encore joli gaçron." The scar that still remained shewed that the wound had been a deep and severe one.

Since I was told this story, I have repeatedly made use of the simple remedy alluded to; and, on more occasions than one, I have had to thank the old soldier for the useful hint he gave me.—*Medico-Chirurgical Review.*

